# Untargeted Metabolomics Reveals Alterations in the Primary Metabolites and Potential Pathways in the Vegetative Growth of Morchella sextelata

**DOI:** 10.3389/fmolb.2021.632341

**Published:** 2021-03-09

**Authors:** Kejun Deng, Xiuhua Lan, Qing Fang, Mengke Li, Guangbo Xie, Liyuan Xie

**Affiliations:** ^1^ School of Life Science and Technology, Center for Informational Biology, University of Electronic Science and Technology of China, Chengdu, China; ^2^ Soil and Fertilizer Research Institute, Sichuan Academy of Agricultural Sciences, Chengdu, China

**Keywords:** Morchella sextelata, untargeted metabolic, primary metabolite, vegetative growth stage, metabolic pathway

## Abstract

*Morchella sextelata,* one of the true morels, has recently been artificially cultivated with stable production in China. Analysis of the variations in primary metabolites during the vegetative stages of *M. sextelata* is critical for understanding the metabolic process. In this study, three developmental stages were categorized based on morphological and developmental changes, including the young mushroom period, marketable mature period, and physiological maturity period. Untargeted metabolomics-based mass spectrometry was used to analyze the change of metabolites during the growth stages of *M. sextelata*. The result showed that the metabolites’ content at the different growth stages were significantly different. The relative contents of linoleic acid, mannitol, oleamide, and betaine were higher at each growth stage. Flavor substances were significantly metabolizable during commodity maturity, while amino acids, organic acids, and lipids were significantly metabolizing at physiological maturity. Pathway analysis of the most significant changes involved Pyrimidine metabolism, Vitamin B6 metabolism, Arginine biosynthesis, Lysine biosynthesis, and Lysine degradation. The results can provide a theoretical basis for further clarifying the metabolic regulation mechanism and lay the foundation for optimizing the cultivation process of *M. sextelata*.

## Introduction

*Morchella sextelata* belongs to the family Morchellaceae, which is a delicious edible and medicinal mushroom documented in the prestigious pharmaceutical classic “Compendium of Materia Medica”, written during the Ming Dynasty of China (Liu et al., 2018; Tietel and Masaphy 2018). Morel was expensive because it used to depend on wild resources. Now, the artificial cultivation of Morchella sp. has been successful through exogenous nutrient bag technology and can obtained with a stable yield. The most commonly cultivated morel is black morel, of which *M. sextelata* has good commercial characteristics of fresh mushrooms. As a result, it has gradually become one of the common nutritional sources for humans.

The genome sequence of *M. sextelata* has already been reported (Han et al., 2019). Genome (Liu et al., 2020a; Liu et al., 2020b), transcriptomes (Liu et al., 2019), and proteomics (Wang et al., 2019) have also been reported in several morels. These findings allowed us to understand the genetics and regulation of Morchella based on the gene and protein. However, there is still a lack of information about their metabolites and how to reflect the growth stages.

Metabolomics is increasingly used as a powerful tool of systems biology following genomics, transcriptomes, and proteomics, which had been introduced into the study of edible fungi (Son et al., 2019). Most studies focus on quality improvement, yield improvement, and temperature stress. The purpose of this study is to conduct an extensive metabolic analysis of *M. sextelata*, and to determine the important metabolites and the specific nutritional characteristics. The results can provide a theoretical basis for optimizing the cultivation process of *M. sextelata* and guide the development and genetic improvement of *M. sextelata*. Additionally, it can help us to understand the metabolic pathway, nutritional composition, and optimizing cultivation process of high-quality morel.

## Materials and Methods

### Plant Growth

The rice field was chosen to build a shed for the cultivation of *M. sextelata*. After harvesting, the straw was smashed and plowed to loosen the soil. During cultivation, soil was kept moist, good ventilation was maintained, and light was “half shade and half Sun” in the shed. About 15–25 days after planting *M. sextelata*, exogenous nutrition bags (100% of straw or 85%–90% of straw and 15%–10% of the husk, mixed with 1% of lime and 1% of gypsum) were used. When the mushroom began to emerge, the temperature in the shed was controlled at 8–20°C, and the relative humidity was kept between 85 and 90%.

The young mushroom had obvious fruiting body characteristics, the cover was smooth, the ridge was not unfolded, the color was light gray or gray-brown, the stipe was white or light yellow white, and the cylinder or the base was slightly expanded. 20–30 days later, the fruit body grew until the height of the cap grew to 3–6 cm, which can be sold in the market. The marketable mature stage occurs 7 days later; the fruiting body is in a physiological mature stage. At this time, the fruiting body’s cap is reddish-brown to dark reddish-brown, the density of the ridge of the cap is medium, and the longitudinal ridge is very obvious. The fruiting layer of the cap is pale-pink and the stipe is smooth, white, and trapezoid.

### Metabolites Extraction

Fresh samples were individually ground with liquid nitrogen and the homogenate (100 mg) was vortexed with pre-chilled methanol (0.1% formic acid). The suspensions were incubated on ice for 5 min and then were centrifuged at 15,000 rpm, 4°C for 5 min. The supernatant was diluted to a final concentration of 60% methanol in LC-MS grade water. Subsequently, the samples were subsequently transferred to a fresh Eppendorf tube with 0.22 μm filter and then were centrifuged at 15,000 g, 4°C for 10 min. Finally, the filtrate was injected into the LC-MS system for analysis.

### LC-MS Analysis

LC-MS analyses were performed using a Vanquish UPLC system (Thermo Fisher) coupled with an Orbitrap Q Exactive HF-X mass spectrometer (Thermo Fisher). Samples were separated onto a Hyperil Gold column C18 (100 × 2.1 mm, 1.9 μm) using a 16-min linear gradient at a flow rate at 0.2 ml/min. The eluents for the positive polarity mode were eluent A (0.1% FA in Water) and eluent B (Methanol). The eluents for the negative polarity mode were eluent A (5 mM ammonium acetate, pH 9.0) and eluent B (Methanol). The solvent gradient was set as follows: 2% B, 1.5 min; 2–100% B, 12.0 min; 100% B, 14.0 min; 100–2% B, 14.1 min; 2% B, 16 min. Q Exactive HF-X mass spectrometer was operated in positive/negative polarity mode with a spray voltage of 3.2 kV, the capillary temperature of 320°C, sheath gas flow rate of 35 arb and aux gas flow rate of 10 arb, and the data-dependent acquisition (DDA) procedure was used to MS/MS scan.

### Data Acquisition

The raw data files generated by UPLC-MS/MS were processed using the Compound Discoverer 3.0 (CD 3.0, Thermo Fisher) to perform peak alignment, peak picking, and quantitation for each metabolite. The main parameters were set as follows: retention time tolerance, 0.2 min; actual mass tolerance, 5ppm; signal intensity tolerance, 30%; signal/noise ratio, three; and minimum intensity 100,000. After that, peak intensities were normalized to the total spectral intensity. The normalized data were used to predict the molecular formula based on additive ions, molecular ion peaks, and fragment ions. And then peaks were matched with the mzCloud (https://www.mzcloud.org/) and ChemSpider (http://www.chemspider.com/) database to obtain the accurate qualitative and relative quantitative results.

### Statistical Analysis

Principal components analysis (PCA) and partial least squares discriminate analysis (PLS-DA) were constructed to distinguish the different growth stages’ samples. The data was mean-centered and Pareto-scaled before PCA and PLS-DA. The variable importance in the projection (VIP) value of the first principal component of the PLS-DA model was used, and the *p* expression of the *t*-test was used to find the differential expression of metabolites. The compound with VIP values >1 in the PLS-DA model were identified as differential metabolites. Furthermore, the generalization ability of fitting models was evaluated by 200 permutations of cross-test validation.

### Pathways Analysis

Pathway Analysis was processed the pathway analysis module of MetaboAnalyst 4.0. Hypergeometric tests and relative betweenness centrality were selected for pathway enrichment analysis and pathway topology analysis.

## Results and Discussion

### Univariate Analysis of Three Growth Stages of *M. Sextelata*

**FIGURE 1:**
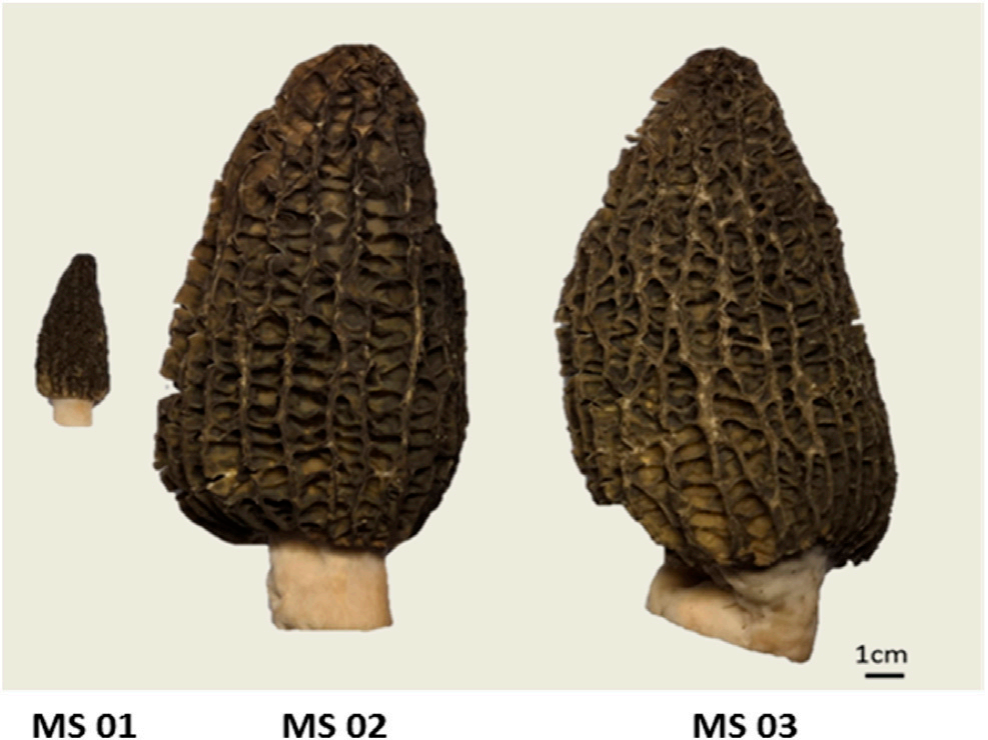
The morphology in three growth stage of M. *sextelate.*

To understand the chemical base of *M. sextelata*, the three different growth stages’ samples (Figure 1), including the young mushroom period (MS 01), marketable mature period (MS 02), and physiological maturity period (MS 03), were selected, and each stage sample had four biological replicates. In total, 292 metabolites were detected in negative ion mode, and 381 metabolites were detected in positive ion mode. Since two datasets were obtained from the same set of samples, in the following our statistical analyses were performed by ion mode for model construction.

To assess the stability of the experiment system and method, three quality-control (QC) samples were used to monitor the stability of the analysis. The QC sample was obtained by mixing equal amounts of each study sample. Univariate analysis was performed between a pair of samples, and Pearson correction was applied to reduce the false positives. A higher correlation of QC samples (from 0.989 to 0.995) indicates better stability of the detection method and higher quality of the analysis data. At the same time, a solvent blank control was set to assess any background carryover in the chromatographic and mass-spectra system.

In the three groups of samples, essential amino acids, such as lysine, methionine, threonine, phenylalanine, tryptophan, valine, leucine, and histidine, which are considered to limit the nutritional quality of plants because their contents in plants are very low compared with the levels required for optimum growth of human and animals, were identified in this study. Among these, threonine and leucine had been reported previously in the literature (Tietel and Masaphy 2018).The top 20 compounds were analyzed in the positive and negative mode, including amino acids, fatty acids, and organic acids (Table 1). In the negative ion mode, linoleic acid, mannitol, and citric acid had the highest response value, which were reported in other morels (Heleno et al., 2013; Tietel and Masaphy 2018). In the positive ion mode, oleamide, betaine, and 5-hydroxymethyl-2-furaldehyde had the highest response value.

**TABLE 1:** The top 20 metabolites in the different growth stages in M. *sextelata*.

Compound name	Formula	Molecular weight	Mode	Average peak area response			Fold change FC	
				MS 01	MS 02	MS 03	MS 02 vs. MS 01	MS 03 vs. MS 02
Amino acid								
D-Glutamine	C5H10N2O3	146.0691	neg	52517779.6	55300117.5	50919710.5	2.95	0.49
			pos	175020630	202852560	197316628	2.84	0.57
Leucine	C6H13NO2	131.0943	neg	27669570.7	30005152.1	21563293.5	1.18	0.37
L-Histidine	C6H9N3O2	155.0691	neg	30631934.5	28965265.6	28497511.4	2.09	0.81
L-Glutamic acid	C5H9NO4	147.0531	neg	24626614.4	25199141.5	22202657.6	0.21	1.18
			pos	73009090.7	71272283.8	70764604.1	1.14	0.75
Betaine	C5H11NO2	117.0789	pos	713858495	738743377	704354527	0.70	0.77
L-Pyroglutamic acid	C5H7NO3	129.0426	pos	174775561	202494097	197194396	2.84	0.57
Arginine	C6H14N4O2	174.1116	pos	93846877.8	132056346	147646909	6.73	1.73
L-Ergothioneine	C9H15N3O2S	229.0882	pos	97394439.2	100689577	100817320	2.82	0.56
Tryptophan	C11H12N2O2	204.0898	pos	68585075.9	75979616.1	69573701.8	0.86	1.15
Valine	C5H11NO2	117.0789	pos	60133122.9	73897445.4	72007330	1.01	0.62
L-Phenylalanine	C9H11NO2	165.079	pos	55561653.3	52859391.3	55756976.7	0.65	0.94
L-Norleucine	C6 H13NO2	131.0946	pos	49003106.5	45036780.7	41573635.5	0.79	0.46
Organic acid								
Citric acid	C6H8O7	192.0269	neg	210288027	200048092	204215239	5.48	0.60
			pos	185711529	156009461	149553605	1.34	0.20
Malic acid	C4H6O5	134.0212	neg	141642631	168105063	156985761	2.18	2.60
α-Ketoglutaric acid	C5H6O5	146.0215	neg	94945828.5	87736412.8	83529313.5	3.79	0.42
			pos	54637424.2	58639473.7	58365152.1	1.33	0.99
Glutaric acid	C5H8O4	132.0416	neg	32329178.7	27022905.1	24824414.7	0.51	5.70
D-Quinic acid	C7 H12 O6	192.0629	neg	30086057.3	25999702.5	24270793.4	0.94	Up
Fumaric acid	C4H4O4	116.0107	neg	13779089.3	16393535.5	15796205	2.37	3.07
Indole-3-acrylic acid	C11H9NO2	187.0632	pos	69205548.6	76637318.5	70566485.1	0.86	1.15
Itaconic acid	C5H6O4	130.0266	pos	73126248.2	71341813.2	70906423.1	1.15	0.75
Nicotinic acid	C6 H5 N O2	123.032	pos	53099588.7	48029261.5	50445649.6	1.50	0.37
Fatty acid								
Linoleic acid	C18H32O2	280.2389	neg	19985778.6	25512206.8	24549325.6	1.01	0.90
Oleic acid	C18H34O2	282.2546	neg	149239267.8	143281500.1	140734758.5	1.43	0.89
5-Aminovaleric acid	C5H11NO2	117.07885	neg	24700318.5	34608349.3	29176979.3	1.84	0.59
2-trans-Hexadecenoic acid	C16 H30O2	254.2236	neg	30466794.19	31518552.6	25998519.8	0.83	0.83
α-Linolenic acid	C18H30O2	278.2237	neg	1175579475	1053632822	1146435322	1.15	0.79
Stearic acid	C18H36O2	284.2706	neg	17234851.2	16017661.2	16485969.9	0.91	0.70
Oleamide	C18H3 NO	281.2712	pos	942923478	964094352	817300096	0.99	0.99
Hexadecanamide	C16H33NO	255.2557	pos	30466794.2	31518552.6	25998519.8	0.99	0.85
Others								
D-Mannitol	C6 H14 O6	182.0784	neg	186785262	218448336	205127331	0.48	0.80
α,α-Trehalose	C12H22O11	342.1150	neg	18912172.1	19883986.9	19265468.3	0.92	0.66
D-Glucose 6-phosphate	C6 H13O9P	260.0286	neg	13541907.1	16070917.2	14931659.9	0.83	0.22
UDP-N-acetylglucosamine	C17H27N3O17 P2	607.0802	neg	19601944.1	15942221.5	14870979.7	0.57	0.32
5-Hydroxymethyl-2-furaldehyde	C6H6O3	126.0316	pos	280980933	318206745	258882136	0.22	1.61
Acetylcholine	C7H15NO2	145.1102	pos	115996491	139822151	129670758	0.95	0.74
Nicotinamide	C6 H6 N2 O	122.048	pos	48422213.8	44450342.3	43397383	0.92	0.46

### PCA and PLS-DA of Three Growth Stages of *M. Sextelata*

**FIGURE 2:**
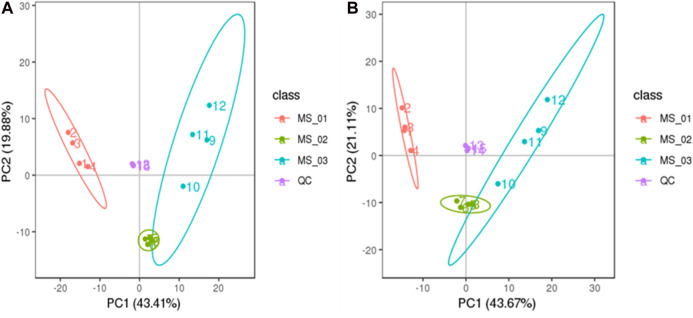
PCA analysis of the three group sample **(A)**: positive ion mode. **(B)**: negative ion mode.

To assess the difference in total metabolisms among the samples of each group and the variation of intra-group samples, 292 metabolites detected in negative ion mode from three growth stages of *M. sextelata* were analyzed in PCA. The first (PC1) and second (PC2) principal components accounted for 43.67% and 21.11% of the total variance, respectively. The growth stages of *M. sextelata* were completely divided into three clusters (Figure 2A). 381 metabolites detected in positive ion mode were similarly processed into three clusters, the first (PC1) and second (PC2) principal components explained 43.41% and 19.88 % of the total variance, respectively (Figure 2B). The results showed that the metabolites in different growth stages of *M. sextelata* were significantly different and metabolomics can be used to study the physiological mechanism of *M. sextelata*.

**FIGURE 3:**
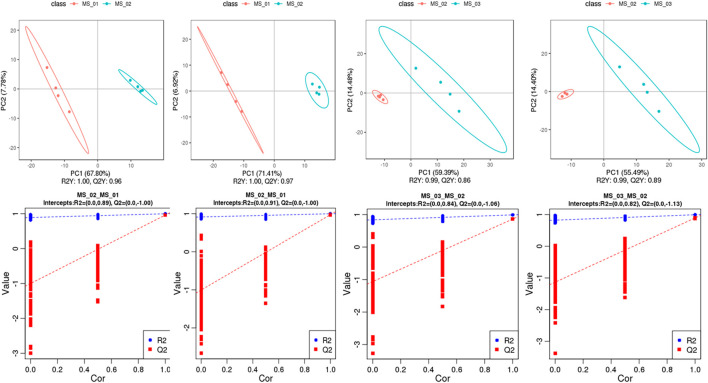
PLS-DA score plots and validation plots. PLS-DA score for MS 02 vs. MS 01 **(A)**, MS 03 vs. MS 02 **(C)** in negative ion mode; MS 02 vs. MS 01 **(B)**, MS 03 vs. MS 02 **(D)** in positive ion mode; validation plots for MS 02 vs. MS 01 **(E)**, MS 03 vs. MS 02 **(G)** in negative ion mode; MS 02 vs. MS 01 **(F)**, MS 03 vs. MS 02 **(H)** in positive ion.

To distinguish the differences of metabolites in the each group, the PLS-DA model between metabolite differences and sample category was established, and the model quality parameters (R2, Q2) were achieved by 7-fold cross-validation and sorting verification (Figure 3). The data to the models (R2Y = 1.00 and Q2 = 0.96 in negative ion mode, R2Y = 1.00 and Q2 = 0.97 in positive ion mode, respectively) indicated excellent fit to explain the sample differences between MS 01 and MS 02 in each comparison group. The cross-validation in those two models (intercepts, R2= (0.0, 0.89) and Q2= (0.0, −1.00), R2= (0.0, −0.91) and Q2= (0.0, −1.00), respectively) revealed no over-fitting to show high predictive capability. The data to the models between MS 02 and MS 03 in each comparison group (R2Y = 0.99 and Q2 = 0.87 in negative ion mode, R2Y = 0.99 and Q2 = 0.89 in positive ion mode, respectively) also showed a clear separation. The cross-validation (intercepts, R2= (0.0, 0.84) and Q2= (0.0, −1.06), R2= (0.0, 0.82) and Q2= (0.0, −1.13), respectively) also showed high predictive capability. These results suggested that the PLS-DA model was reliable and can be used to identify the different metabolites, consistent with the PCA result.

### Identification of Differential Metabolites in Three Growth Stage of M. Sextelata

To identify the differentially expressed metabolites among the three growth stages of *M. sextelata*, the first principal component of variable importance projection (VIP) of the PLS-DA model and *p*-value of *t*-test were used. The fold change value (FC) for each differential metabolite was transformed as Log2, and the corresponding *p*-value was transformed as -Log10. The threshold was set to VIP >1.0, the difference multiple FC > 2.0 or FC < 0.5, and *p*-value < 0.05. Volcano plots were generated based on the FC and the *p* value. Finally, the relative quantification values of differential metabolite were z-transformed for hierarchical clustering analysis (HCA).

**FIGURE 4:**
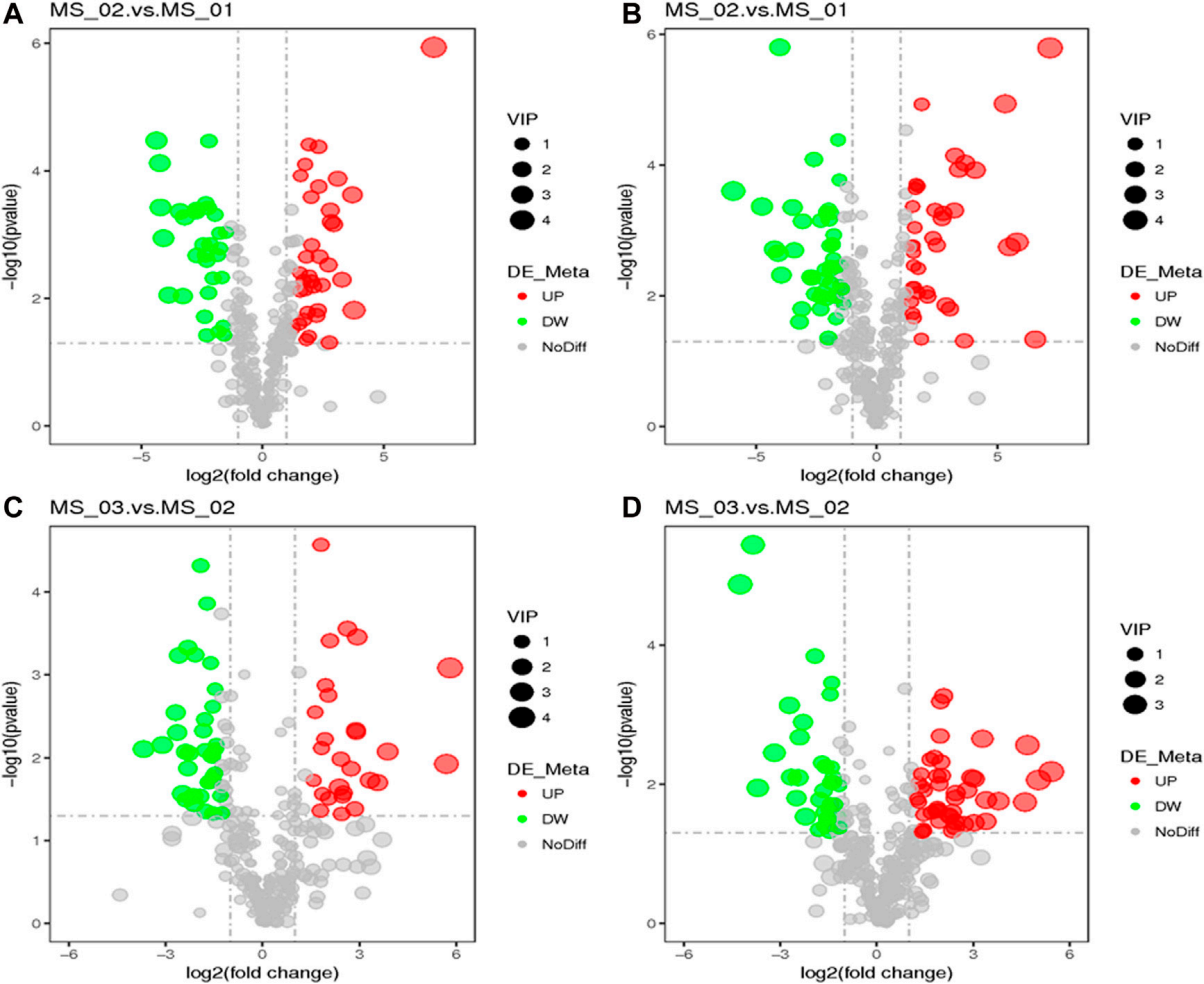
A volcano plot for the differential metabolites. MS 02 vs. MS 01 **(A)**, MS 03 vs. MS 02 **(C)** in negative ion mode; MS 02 vs. MS 01 **(B)**, MS 03 vs. MS 02 **(D)** in positive ion mode.

**FIGURE 5:**
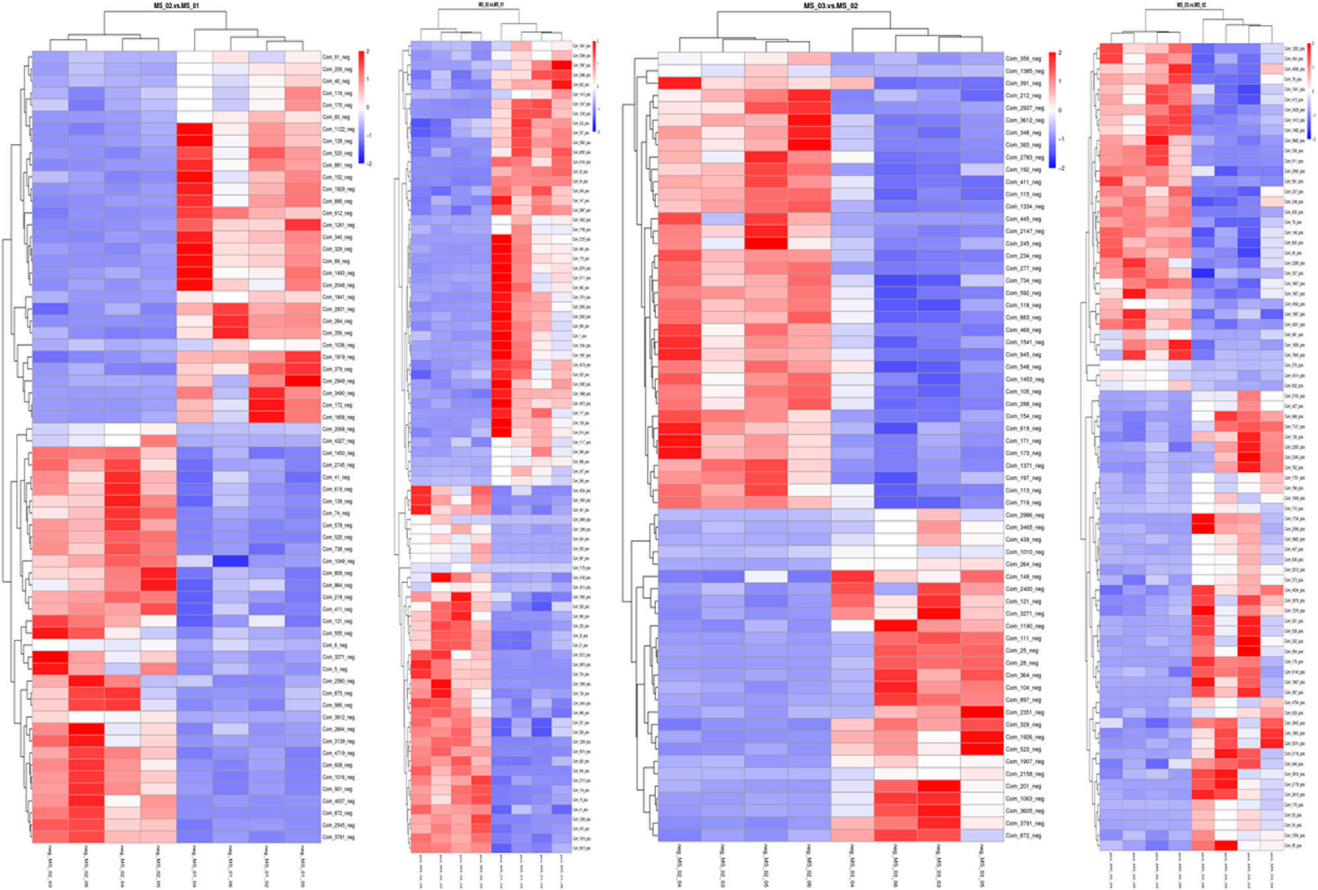
Heatmap of the different metabolites between the samples. Metabolites indicated in red and blue were upregulated and downregulated, respectively. **(A)**: MS 02 and MS01 in negative ion mode; **(B)**: MS02 and MS01 in positive ion mode; **(C)**: MS 03 and MS 02 in negative ion mode, **(D)**: MS 03 and MS 02 in positive ion mode.

According to these rules, 292 (negative ion mode) and 381 (positive ion mode) metabolites were screened out. There were 66 significantly different metabolites between MS 02 and MS 01 (35 up-regulated, 31 down-regulated) and 64 between MS 03 and MS 02 (27 up-regulated, 37 down-regulated) from negative ion modes. There were 84 significantly different metabolites between MS 02 and MS 01 (38 up-regulated, 46 down-regulated) and 79 between MS 03 and MS 02 (34 up-regulated, 45 down-regulated) from positive ion modes (Supplementary Table S1). The volcano plots between each comparison group exhibited the differential metabolites that contributed to the sample separation. (Figure 4). Hierarchical clustering heat map showed distinguishable metabolites related to different metabolic pathways (Figure 5).

In total, at the marketable maturity stage (MS02), the relatively high content of metabolites such as D-glutamine, arginine, L-aspartic acid, L-pyroglutamic acid, L-ergothioneine, and α-ketoglutaric acid increased significantly compared with the young mushroom stage (MS01), which related to arginine biosynthesis and citrate cycle. The result showed there was significant nitrogen metabolic and energy metabolism activity from the young to the marketable maturity stage. Compared with the physiological maturity stage (MS03), the relatively high content of metabolites, such as D-glucose 6-phosphate, nicotinic acid, and UDP-N-acetylglucosamine, which related to nitrogen metabolism and vitamin B6 metabolism, indicated a significant decrease. But D-quinic acid and glutaric acid were significantly increased, which related to lysine degradation.

### Metabolic Pathway Analysis

**FIGURE 6:**
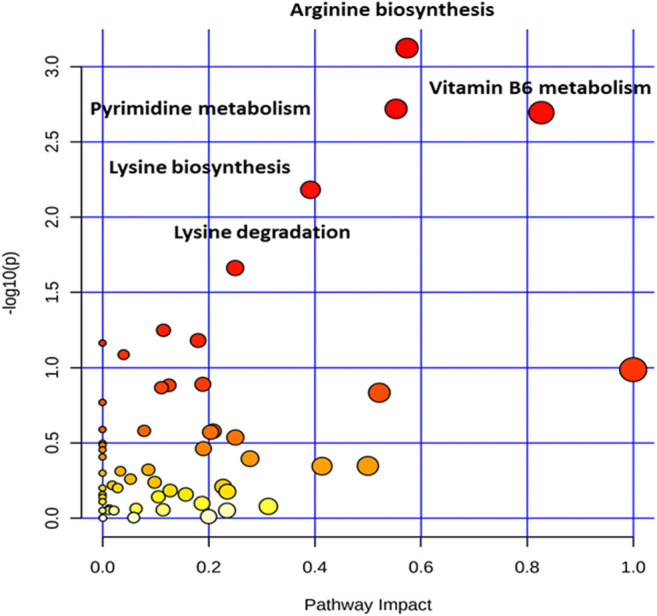
The pathway impact analysis. The metabolic pathways were represented as circles according to their scores from enrichment (y-axis) and topology analyses (pathway impact, x-axis) using MetaboAnalyst 4.0. Darker circle colors indicated more significant changes of metabolites in the corresponding pathway. The size of the circle corresponds to the pathway impact score and was correlated with the centrality of the involved metabolites. Pathways were annotated by numbering when the *p* values calculated form the enrichment analysis were 0.05.

To further explore the metabolic pathways involved in the significantly different metabolites, the identified compounds were annotated with KEGG and the metabolic pathway was analyzed by metaboAnalyst4.0 (https://www.metaboanalyst.ca/) (Chong et al., 2018). The “*Arabidopsis thaliana* (thale cress) (KEGG)” library was selected to perform pathway enrichment analysis and pathway topology analysis based on a hypergeometric test and relative-betweenness centrality, respectively. The results of the metabolic pathway analysis were presented in a bubble plot; each bubble corresponded to a metabolic pathway, and the size of the bubble represented the level of enrichment (Figure 6).

**FIGURE 7:**
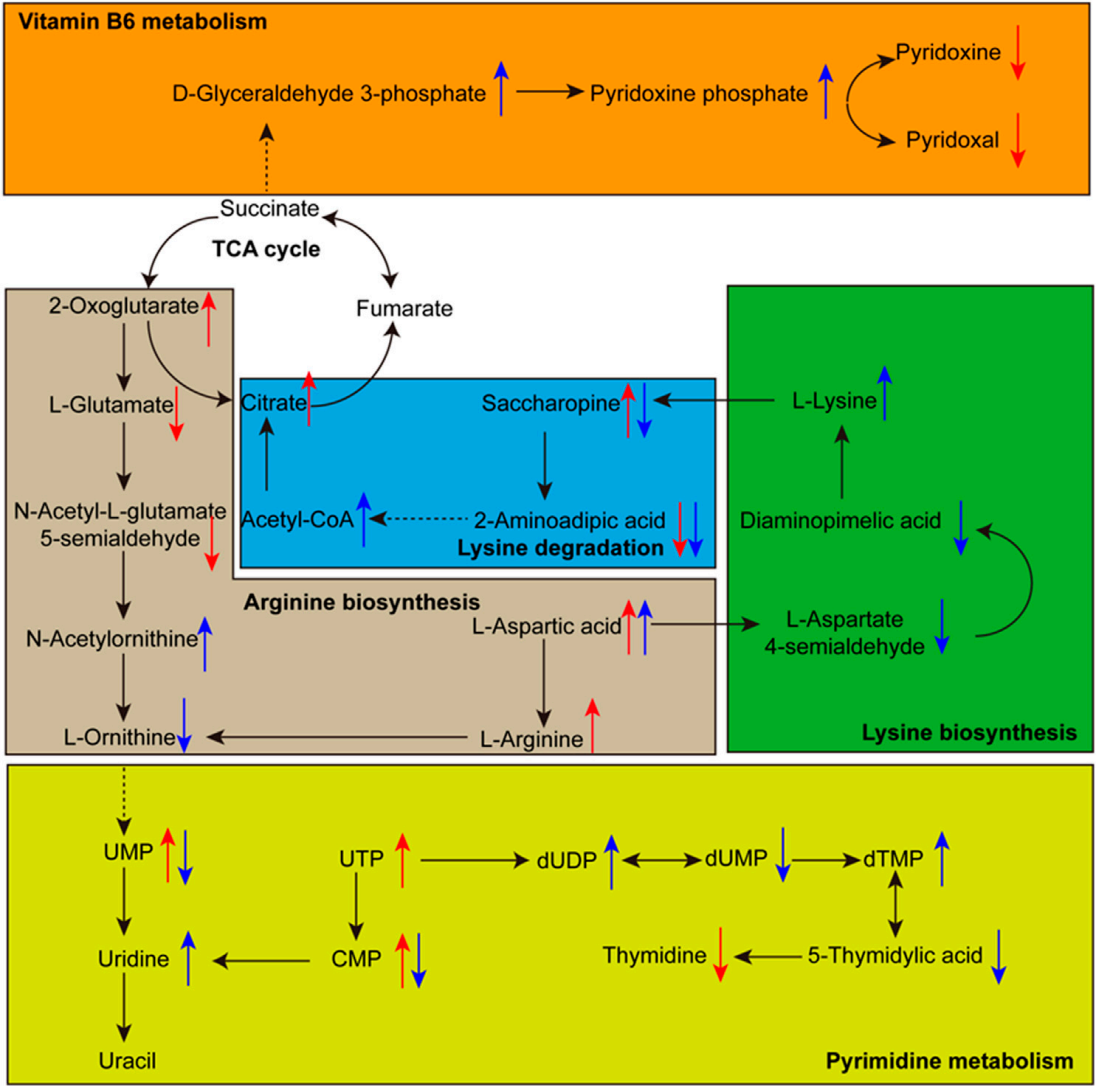
The relationship among some significantly changed pathways and differential metabolites involved in each metabolic pathway. The red arrow indicates the growth stage from young mushroom to marketable mature mushroom, the blue arrow indicates the growth stage from marketable mature mushroom to physiological maturity mushroom. The directions of the arrow indicate the up-regulation and down-regulation of metabolites.

Five main metabolic pathways for differential metabolites were sorted out with a P-value less than 0.05 (Supplementary Table S2), including pyrimidine metabolism, vitamin B6 metabolism, arginine biosynthesis, lysine biosynthesis, and lysine degradation (Figure 7). These pathways mainly focused on energy and nitrogen metabolism.

#### Pyrimidine Metabolism

Pyrimidine metabolism is a form of nucleotide metabolism and plays an essential role in bio-energetic processes and in the synthesis of transport forms of carbohydrates like sucrose, cellulose, most cell wall matrix polysaccharides, and sugar (Stasolla et al., 2003; Zrenner et al., 2006). Ten compounds in the Pyrimidine metabolism pathway were annotated. Among them, UMP in the marketable mature stage is upregulated by 4.94-fold compared to the young mushroom period and downregulated by 1.32-fold compared to the physiological maturity period. UMP in the plant is a feedback inhibitor of carbamoyl phosphate synthetase, but this inhibition is overcome by ornithine, which leads to carbamoyl phosphate being used for arginine biosynthesis in the morels (Zrenner et al., 2006).

#### Arginine Metabolism

Arginine metabolism plays a key role in nitrogen distribution and recycling in plants (Winter et al., 2015). Arginine is the synthesis of glutamic acid from ornithine and then arginine from ornithine. In the marketable mature stage, glutamic acid is downregulated by 2.76-fold compared to the young mushroom period, ornithine is downregulated by 2.76-fold compared to the physiological maturity period, and arginine is upregulated by 2.03-fold compared to the young mushroom period, respectively. Due to the highest nitrogen to carbon ratio, arginine was considered suitable as a storage form of organic nitrogen, an essential metabolite for many cellular and developmental processes (Winter et al., 2015).

#### Lysine Biosynthesis and Lysine Degradation

Lysine biosynthesis and lysine degradation also play an important role in the regulation of nitrogen balance and energy metabolism in plant morels (Stasolla et al., 2003; Zrenner et al., 2006). Lysine belongs to the aspartate family pathway, which is upregulated by 1.88-fold in the physiological maturity period. The content of L-aspartic acid increased beginning with the young mushroom period. But L-aspartic 4-semialdehyde is downregulated by 1.37-fold in the marketable mature period and there is no change in the physiological maturity period. Diaminopimelic acid, g-butyrobetaine, saccharopine, 2-aminoadipic acid, and D-sedoheptulose 7-phosphate showed an obvious change trend. The aspartate family pathway connected with tricarboxylic acid cycle (TCA), enhancing lysine synthesis and inhibiting its catabolism, had a notable influence on the levels of several TCA metabolites in Arabidopsis (Azevedo and Lea 2001; Yang et al., 2020). In this experiment, the same effect was observed. Citric acid and alpha-ketoglutaric acid were upregulated by 1.69-fold and 1.20-fold from the young mushroom to the marketable mature period, respectively. Acetyl-CoA was upregulated by 1.73-fold in the physiological maturity period compared with the marketable mature period.

#### Vitamin B6 Metabolism

Vitamin B6 metabolism is a cofactor and form of vitamin metabolism. Among Vitamin B6, pyridoxine (PN) and pyridoxal (PL) were downregulated by 3.21-fold and 1.29-fold in the marketable mature period. Pyridoxal 5'-phosphate (PLP), which is the most important vitamin B6 and serves as the cofactor for numerous proteins and enzymes, was upregulated by 1.59-fold in the physiological maturity period. In the recent report, there was an unanticipated link between vitamin B6 homeostasis and nitrogen metabolism (Rosenberg et al., 2017). With the same change trend, D-xylulose 5-phosphate is upregulated by 1.29-fold in the physiological maturity period. But glutamine and glyceraldehyde 3-phosphate had no distinct change.

A large number of carbohydrate synthetases were found in *M. importuna*. (Liu et al., 2019; Wang et al., 2019). During the growth process in *M. importuna*, carbon sources needed the supply of external nutrient bags, but no significant acquisition of exogenous nitrogen was observed (Tan et al., 2019). In this study, annotation analysis of metabolites with obvious differences in the growth process shows that there are obvious metabolic differences in amino acid metabolism related to nitrogen sources, which maintained a delicate balance during vegetative growth in *M. sextelata*. It has been reported that the morel spore can be used to increase crop yields (Phanpadith et al., 2020), however whether there is a relationship with the presence of large amounts of nitrogen in Morchella sp. still needs further study.

## Conclusion

In the present work, an untargeted metabolomics method based on liquid chromatography coupled with tandem mass spectrometry (LC-MS) was conducted to rapidly identify primary metabolic changes in the vegetative growth process of *M. sextelata*. Using this approach, the compounds with the top 20 response values included 12 amino acids, nine fatty acids, and eight organic acids. Among them, eight essential amino acids including lysine, methionine, threonine, phenylalanine, tryptophan, valine, leucine, and histidine were identified in this study. The differential metabolites in three growth stages of *M. sextelata* were screened out and analyzed for their metabolic pathways, which were mainly involved in Pyrimidine metabolism, Vitamin B6 metabolism, Arginine biosynthesis, Lysine biosynthesis, and Lysine degradation. The results can provide a strategy to further explain the metabolic regulation mechanism in the vegetable growth stage and provide the foundation for special-nutrition breeding of *M. sextelata*.

## Data Availability

The original contributions presented in the study are included in the article/Supplementary Material, further inquiries can be directed to the corresponding authors.
